# Development of an American and Australian co-designed youth mental health literacy program

**DOI:** 10.3389/frcha.2022.1018173

**Published:** 2023-06-01

**Authors:** Christine Grové, Alexandra Marinucci, Joanne Riebschleger

**Affiliations:** ^1^Monash University, Melbourne, VIC, Australia; ^2^Michigan State University, East Lansing, MI, United States

**Keywords:** mental health, adolescents, school based program, mental illness, youth, co-design, mental health literacy

## Abstract

Adolescence is marked by a high prevalence of mental health concerns, with approximately 14% of young individuals receiving a diagnosis of a mental illness disorder. This figure is projected to rise in the future. However, barriers such as limited access to mental health services, a shortage of mental health professionals, and the enduring stigma surrounding mental health prevent many adolescents from seeking help, potentially resulting in long-term negative outcomes. To address these challenges, an evidence-based mental health literacy program implemented within schools offers a promising avenue for imparting knowledge and improving adolescents' mental well-being. This paper presents a mental health literacy and action program specifically tailored for adolescents, developed in collaboration with professionals, teachers, parents, and adolescents themselves. Lessons learned from program development and implementation in Australia and the United States are shared, providing insights into the process of designing and executing such programs. By enhancing mental health literacy and promoting help-seeking behaviors, this program has the potential to facilitate positive changes in adolescents' mental health outcomes.

## Introduction

In youth ages 11–14, untreated mental health problems such as anxiety, depression, and other disorders (1) are often unremitting into adulthood, and are associated with a trajectory of short- and long-term distress, impairment, costs, and burden (2–4). For decades, mental health problems in children are under-diagnosed and undertreated (5–9). Youth recognition of possible mental health problems and knowledge about appropriate treatment are important foundations to facilitate treatment-seeking actions for self or others (10, 11). However, youth overall knowledge of mental health, also called mental health literacy, is quite low (12). Their ideas about mental health are often drawn from stigmatised media portrays of mental illness where people with mental illness are shown as violent and incompetent (13).

Mental health literacy (MHL) interventions are often designed to provide accurate, non-stigmatised information to people who are then empowered with mental health knowledge that they can use to maintain their mental health or prevent, delay, or reduce the severity of mental health disorders. Mental health literacy interventions have five components, including: (1) understanding mental health; (2) describing ongoing recovery with mental illness; (3) giving examples of mental illness stigma, with response strategies; (4) seeking help for oneself when mental health illness symptoms arise; and (5) seeking help with others living with mental health disorders, especially when the person displays risk of self-harm or seems unable to engage in activities of daily living (14, 15).

There are numerous studies demonstrating that when adults are given accurate information about mental health they are more likely to seek help earlier, use mental health services more often, and identify ways to reduce negative impacts of mental health symptoms (16, 17). Public mental health literacy about mental health problems in children and adolescents is lacking. This can delay or prevent access to critical treatment. Parents, teachers and youth themselves are central agents in advocating and seeking help, yet little is known about youth mental health literacy needs or the extent to which they know to take actions to respond to mental health symptoms, stigma, and stress (18–20). Accurate mental health literacy in parents and teachers about youths' psychological problems may play a pivotal role in being able to navigate treatment access in an underfunded and under-resourced system (21–23).

Mental health disorders are associated with negative secondary outcomes, including reduced educational achievement, poor social functioning, school refusal, and substance misuse (24–27). Despite rising rates of mental health issues among children and adolescents, it appears that there is limited knowledge about how to help youth obtain and use accurate, non-stigmatised mental health information.

## Youth mental health literacy

Mental health literacy can be viewed as a foundation for mental health promotion, early intervention for mental illness and a way for children and youth to link with necessary supports (28–30). The present study examines the MHL Child Focused Model proposed by Bale et al. (31), which aims to elucidate the applicability of Mental Health Literacy (MHL) principles to the domain of children and youth. Through a Delphi study involving professionals in the field of child and youth mental health, such as psychologists, psychiatrists, researchers and teachers, the model was devised. This model comprises six principal components, each encompassing specific subcomponents that highlight areas of focus of child mental health. The components include the recognition of mental health changes, actions related to seeking help, available support systems, influential factors affecting mental health, coping mechanisms and resilience, and attitudes toward mental health (31). By delineating the child and youth-specific elements of MHL, targeted interventions can be designed to address the diverse facets needed to support illness prevention.

Schools are identified as an important setting for MHL programs due to direct access to children and youth, the setting to apply and learn skills is already established and schools play an important role in the health, social and emotional development of students (32–35). Clarke (36) suggests the following are key characteristics for effective programs set within a school environment: (a) universal whole school approach with curriculum embedded within school, (b) skills to enhance social and emotional competencies, (c) strong theory base and well-designed goals, (d) competence enhancement strategies, (e) beginning with younger children and progressing throughout grades, (f) explicit implementation guidelines such as a manual, and (g) teacher or parent training. Other characteristics suggested include use of valid and standardized measures to evaluate outcomes, adequate training of teachers and parents in understanding youth mental health, multiple modalities of interventions, and tailoring interventions to suit student needs (37). Training in mental health promotion for all school personnel such as teacher aides, administrators, janitors, and those who interact with students would be beneficial. Mental health first aid is another program that can be used to train the wider school community in how to identify understand and respond to signs of mental illness and substance abuse disorders or how to respond in a “mental health crisis, until appropriate professional help is received or the crisis resolves” [(38), p. 2]. MHL focuses on the development of accurate mental health knowledge and understanding of mental health to obtain and maintain good mental health, which can be cultivated and continues to grow over time (31, 39–41). Awareness of the need for MHL in the school setting is growing however more research is needed to determine the most effective and sustainable way to provide these programs (42, 43). Additional research may further seek to determine the public mental health impact of such programs offered in schools including their structure, acceptance in the school and by the wider community, uptake, and maintenance.

Mental health should be embedded within the school curriculum and culture, involving teachers, students, parents, and communities with a student-centered approach (44–47). Compared to urban, and especially suburban areas, rural people have higher rates of mental illness, suicide, and substance abuse; services are likely to be distant and difficult to access (48).

A recent scoping review of school based MHL interventions for children and youth found many studies did not use a control group for comparison of outcome effects and did not use reliable or valid measures for outcomes (37). Additionally, many studies did not complete follow up measures of outcomes for sustained effects. Limitations of such studies include uncertainty of whether (a) the reported effects are due to the intervention or external variables, (b) the chosen questionnaires measure the outcomes of interest and (c) how the effects change or are sustained over time (49). These limitations pose barriers for widespread dissemination of interventions and funding opportunities (50). The major focus of included studies in this review were recognition of mental ill health; understanding help and support available to self/others; coping and resilience strategies; and knowledge of mental illness stigma. Studies also focused on the importance of positive mental health. The review highlights the critical need for rigorous development and implementation of school based MHL interventions to address the increasing need for preventative approaches to youth mental health.

In response to a clear need for increased mental health literacy among youth, Riebschleger et al. (13, 51) created a program to deliver accurate, non-stigmatised information about mental illness to youth ages 11–15. The program is called *The Youth Education and Support Program (YES)*. There was an emphasis on viewing mental illness from the standpoint of children and youth and not from a traditional diagnostic focused expert perspective (51). Youth perspectives were drawn from studies asking youth their experiences with mental illness among friends and family members and what they wanted to know about mental illness (52, 53). Data were also extracted from studies about child mental health information needs with educators, school-based mental health prevention program staff, mental health professionals, parents, and child welfare professionals (13, 15, 51, 54–56). These data points informed the development and implementation of YES, essentially focusing on youth perspectives while addressing the Jorm (14, 57) five-construct model of mental health literacy. Riebschleger et al. (51) added a sixth construct that included youth views of what it is like to live with a family member with a serious mental illness.

## Development and implementation of YES in the USA

In the USA, initially YES was trialed after school in community mental health settings (15). However this lead to inconsistent child attendance despite providing transportation and incentives such as snacks. Thus, the research team approached an urban school system in Michigan to pilot test an in-school model of *YES* that essentially comprised of youth psychoeducation program plus support for youth management of stress skill building. Youth participants actively engaged in providing feedback for improving the program session by session and end of session written recommendations, and an end of program written evaluation and debrief discussion with the youth. The program was continually revised to respond to youth and expert stakeholder feedback.

Forty-six of 63 YES participants provided pre, end of program, and 2–3 month post intervention data on knowledge of mental illness and use of coping with stress strategies (15). The sample was comprised of 6–8th grade students that were 63% female and 51% diverse (Black, Latinx, mixed race). Over half reported having a relative with a mental illness and 28% of the youth reported having a mental health challenge. Youth knowledge of mental illness increased from pre- to end of intervention (*p* ≤ 0.0001) and the knowledge increase was holding at 2–3 months post intervention. Ninety percent of youth reported increased use of coping strategies to manage their stress.

Over time, what developed was a model that was co-led by a university child clinician/researcher and a school social worker with small groups (about 10–12 youth per group). The youth were ages 11–15. The program facilitators used hands on, interactive exercises and discussions to deliver ten sessions of information about mental illness, recovery, depression/anxiety, substance abuse, co-occurring disorders (mental illness/substance abuse), family experiences with mental illness, crisis planning, stress management strategies, hope for the future, and “graduation.” Stress management check-ins were integrated into every session per youth recommendations. A new scale for measuring youth knowledge of mental illness was developed, as well as a fidelity measure completed by trained observers (15, 58). Findings indicated that youth significantly increased their mental health literacy levels from pre to post intervention; the increase in MHL levels was still holding at 2–3 months post intervention (15). The Riebschleger et al. (15) study also reported that youth attendance was over 90%; the fidelity outcome was 88%, meaning the rate of program content on target with program manual instruction. All of the youth stated they liked the program; several asked to take the program again. All of the youth participants reported somewhat to a great deal of improvement in their ability to manage stress as drawn from a Likert-type individual rating scale. Several youth reported helping family/friends who reportedly told them that they were feeling suicidal to obtain health and mental health crisis services. One had a parent with mental illness; he told a group of international visitors that the best thing about the program was “we learned that mental illness is not our fault.”

## Importance of key stakeholder collaboration

Collaboration among key stakeholders, such as young people, parents, educators and mental health professionals, is recommended when developing and implementing mental health interventions (46, 59–61). The World Health Organization (62) states that collaboration must be initiated from the beginning of development of such interventions as this can contribute to wider acceptability from the target population and feasibility of the intervention under real world conditions (62, 63). *The Youth Education and Support (YES) program* was developed over a period of 12 years based on literature of mental health literacy, existing programs, consultation with mental health experts, parents and young people (13, 15, 54, 56, 58).

## Development and implementation of YES in Australia

The Australian version of the YES program has been adapted by psychologists in the field of educational and developmental psychology with extensive experience working with young people's development and within school environments. The Australian adapted YES program is based on the MHL Child Focused Model as developed by Bale et al. (31). Additionally, the program has been shaped by feedback from and perspectives of young people, teachers, health professionals and school wellbeing staff in the Australia context. To increase sustainability and widespread dissemination, the Australian adapted YES program was assessed for how it aligned with the Victorian Health and Physical Education curriculum (64). The program aligns with the focus area titled, “Mental health and wellbeing”, as part of the Health and Physical Education curriculum. As substance misuse (e.g., alcohol and other drug use) is addressed in health education (65), the Australian adapted version of YES omitted this content and instead emphasizes help-seeking actions, coping skills, and resilience strategies according to the child mental health literacy model developed by Bale et al. (31). Resilience for young people is generally conceptualized as the ability to use problem solving skills, to have a sense of autonomy and purpose, and to cope in times of adversity (15, 66). Resilience, coping, attitudes toward mental health and help seeking are related (67), therefore these were focused on in the Australian adapted YES program. Evidence indicates that young people want to learn about mental health and coping strategies at school (23, 68). The Mission Australian Youth Survey Report (2020) stated that young people were most concerned about coping with stress and mental health in the past year (and during COVID-19). Many young people do not know where to seek help for mental health problems or how to help peers who experience mental illness, presenting a significant barrier for support (19, 69, 70).

## Overall aim of YES in Australia

The Australian Adapted YES Program is called The Youth Mental Health Literacy and Action Program to differentiate between the US and Australian versions. The overall aims of the Youth Mental Health Literacy and Action Program are to increase mental health literacy, appropriate help seeking behavior and resilience, and decrease mental illness stigmatization. The school-based program takes a preventative approach and aims to promote the key components of mental health and give young people tools to manage challenges and look after their own mental health. It specifically focuses on coping strategies, mental health (including recognition of illness, recovery and fostering positive mental health), building resilience, how to seek help and knowledge of supports available, mental illness stigma, and the impact of mental illness on families. The program also includes value and goal exploration to foster self-reflection and awareness. Table 1 outlines the topic and learning objectives of each session of the Australian Adapted YES program. Youth mental health literacy outcomes are measured at pre, post, and at three month follow-up post intervention.

**Table 1:** Comparison between US YES and Australia YES content.

**Session**	**Topic and Learning Objectives**	
	**Australia**	**US**
1	Introduction to YES Þ Understand what to expect in the YES program Þ Describe stress (physical sensations, triggers) Þ Develop group values and expectations during the program	Introduction to YES Þ Describe the YES program and what to expect Þ Describe what stress means and what kinds of situations may trigger it Þ Identify where we feel stress on our bodies when it occurs Þ Develop the rules of the group
2	Introduction to coping Þ Describe coping behaviors Þ Create a coping behavior plan	Coping Þ Describe specific coping behaviors Þ Making a coping behavior plan
3	Mental illness and recovery Þ Understand definition and prevalence of mental illness Þ Understand heritability and recovery processes for mental illness	Mental Illness and recovery Þ Be able to give a definition of mental illness Þ Say that mental illness affect 1 in 5 people Þ State that mental illness is no one's fault Þ Describe how recovery works and how to get help Þ Suggest how to deal with suicidal behaviors
4	Depression and anxiety Þ Understand emotions and behavior associated with depression and anxiety Þ Learn some strategies to manage depressive and/or anxious symptoms	Depression and anxiety Þ Describe how a depressed person may feel and behave Þ Identify the heritability of mental illness including depression Þ Know to tell adults immediately if they or a friend are feeling suicidal Þ Describe how to cope with depression appropriately Þ Describe the recovery process
5	Coping and resilience Þ Develop individual coping strategies Þ Understand healthy behaviors to promote resilience and coping	Substance abuse and addiction Þ Describe how peers may try to pressure them into using illicit substances Þ Relay how substance use can lead to addiction Þ List the type of substances that can be abused Þ Say what drugs and alcohol do to the body and brain Þ Describe that there are greater risks on teenage brains
6	Help seeking and support Þ Understand where to seek help (formal and informal) Þ Understand how to seek help (e.g. helplines, community, professional) Þ Understand how to help others who may experience mental illness Þ Understand process of therapy and what to expect	Co-occurring Þ Define the co-occurrence of mental illnesses, especially substance abuse and depression Þ State the helpful and harmful uses of medication Þ Understand the impact of co-occurring mental illness have on family members Þ Tell why quitting cold-turkey doesn't always work and the influence of withdrawals Þ Describe what goes on in treatment for substance abuse Þ State that substance abuse is a disease and is no one's fault
7	Stigma Þ Understand stigmatised views of people with mental illness Þ Understand how stigmatised people are treated	Stigma Þ Understand the stigmatised views of people with mental illnesses Þ Understand the stigmatised views of people with substance abuse problems Þ Understand how stigmatised people are treated by others
8	Families Þ Understand how mental illness and stigma can affect families	Families Þ Describe how mental illness and substance abuse affects families Þ Identify how stigma affects families Þ Describe good and bad days in the family
9	Remember and hope Þ Exploring values Þ Goal setting directed by values Þ Demonstrate understanding of mental health literacy	Remember and hope Þ Correctly answer 6 or more mental health literacy questions Þ Identify a future goal Þ Identify something they can do to reach the goal Þ Participate in planning for session 10 graduation
10	Graduation Þ Describe what learnt during YES Þ Reflect on coping skills Þ Describe how will use YES learning in future	Graduation Þ Describe what they learned in the YES program Þ Compare their coping skill levels from pre to post Þ Say goodbye to their program participation Þ Describe how they think they will use the YES program learning in the future

The research protocol for the evaluation of the Australian adapted YES program is available (71). This evaluation is a cluster-controlled trial of the program within secondary schools in Victoria, Australia. The trial has been registered on the Australian New Zealand Clinical Trials Registry (Trial number: ACTRN12621000325808). Participants in the control group will complete questionnaires at two timepoints with a 10 week interval and will not receive the YES program. Interested schools in Victoria, Australia will be allocated to the Australian adapted YES program. Students in grades seven to ten will opt into the program and participate at their own school during wellbeing classes, homeroom time or after school (dependent on school preference). Psychologists in training will deliver the program after undergoing program specific training and receive regular supervision by the research team. Measures of MHL, help seeking and resilience are taken pre-program, post-program, and follow up measures are taken three months and one year after completing the program (51, 58). The fidelity scale was essential to improving the program overtime, ensuring what occurred during the sessions matched the session outcomes, aims and content. Both programs attained high scores of fidelity, ranging from 80% for the Australian adapted version of YES and 100% fidelity when YES was implemented in America.

Participants will be asked to complete feedback questionnaires each session and at the end of the program to include their perspectives in future development to meet the needs of young people. With student assent, parents are provided with information each session relevant to the content covered and schoolteachers and/or counselors may sit in on sessions. These key stakeholders will be encouraged to provide feedback for a collaborative and holistic approach to supporting youth mental health and wellbeing. In the USA resources and education material tend to vary among states thus the questionnaires may need to be tailored to the context they will be used. A teacher component to the Australian adapted YES program is currently in development with the aim to (a) educate school teachers of the critical need for MHL within the classroom, (b) increase the competency of school teachers in supporting their students' mental health and wellbeing, and (c) provide practical tools for including MHL in the wellbeing curriculum. These aims have been selected based on feedback from school staff highlighting a lack of understanding and confidence in supporting student mental health (72–74).

## Conclusion

The present study provides an overview of the YES program, which aims to enhance the mental health literacy and actions of the upcoming generation of young individuals. This article provides the program's content and outlines the ongoing efforts to implement and evaluate the YES program, which encompasses co-designed, educational, and evidence-based resources designed to support youth mental health. Notably, the program incorporates valuable input from key stakeholders, including educators, parents/family members, and youth themselves, ensuring its relevance and ongoing effectiveness.

The YES program aligns with the existing body of literature emphasizing the pressing need for MHL programs to address the escalating mental health demands and the low levels of mental health literacy observed among youth. Specifically, the program targets schools as a vital starting point to address these concerns. Should the anticipated outcomes of the pilot study be realized, future research endeavors should involve replicative studies, hoping to progress toward clinical trials.

Advancements in youth MHL, particularly when supported by data derived from replicated studies, hold the potential to establish a robust MHL practice resource within educational settings. This, in turn, can inform policymakers regarding the necessity of supporting such programs and advocate for continued MHL program training among professionals working with youth in schools, health/mental health agencies, and community organizations.

## Author contributions

CG: substantial contributions to the conception or design of the work and agree to be accountable for all aspects of the work in ensuring that questions related to the accuracy or integrity of any part of the work are appropriately investigated and resolved. CG, AM, and JR: drafting the work or revising it critically for important intellectual content and provide approval for publication of the content. All authors contributed to the article and approved the submitted version.
